# Challenges in Meeting Healthy People 2020 Objectives for Cancer-Related Preventive Services, National Health Interview Survey, 2008 and 2010

**DOI:** 10.5888/pcd11.130174

**Published:** 2014-02-27

**Authors:** Martin L. Brown, Carrie N. Klabunde, Kathy A. Cronin, Mary C. White, Lisa C. Richardson, Timothy S. McNeel

**Affiliations:** Author Affiliations: Martin L. Brown, Kathy A. Cronin, National Cancer Institute, Bethesda, Maryland; Mary C. White, Lisa C. Richardson, Centers for Disease Control and Prevention, Atlanta, Georgia; Timothy S. McNeel, Information Management Services, Inc, Calverton, Maryland.

## Abstract

**Introduction:**

Healthy People (HP) is the US program that formulates and tracks national health objectives for the nation. The National Health Interview Survey (NHIS) is a designated data source for setting and evaluating several HP targets in cancer. We used data from the 2008 and 2010 NHIS to provide a benchmark for national performance toward meeting HP 2020 cancer-related objectives.

**Methods:**

HP 2020 cancer screening, provider counseling, and health care access objectives were selected. For each objective, NHIS measures for the overall population and several sociodemographic subgroups were calculated; the findings were compared with established HP 2020 targets.

**Results:**

From 2008 to 2010, rates of breast and cervical cancer screening declined slightly while colorectal cancer screening rates increased by 7 percentage points. Rates of cancer screening and provider counseling were below HP targets. Meeting HP targets seems less likely for subgroups characterized by low income, no health insurance, or no usual source of care. Meeting HP targets for access to health services will require an increase of 18 percentage points in the proportion of persons under age 65 with health insurance coverage and an increase of 10 percentage points in the proportion aged 18 to 64 with a usual source of care.

**Conclusion:**

Whether HP objectives for cancer screening and health care access are met may depend on implementation of health care reform measures that improve access to and coordination of care. Better integration of clinical health care and community-based efforts for delivering high-quality screening and treatment services and elimination of health disparities are also needed.

## Introduction

Healthy People (HP), a program first initiated in 1979 as a Surgeon General’s report (1), tracks 10-year national objectives for improving the health of all Americans. It provides a framework around which public health agencies, private organizations, and the general public can take action. This framework is couched as a series of objectives focused on improvements in a wide variety of indicators for health status, risk reduction and prevention, health service delivery, and surveillance and evaluation. The objectives include specific metrics, or “targets,” that allow progress to be assessed (2).

HP calls for a reduction in cancer deaths overall and in the prevalence of specific cancers (cervical, breast, colorectal, and prostate) that may be reduced by the use of screening tests and the timely and appropriate treatment of early-stage cancers. The National Cancer Institute (NCI) and Centers for Disease Control and Prevention (CDC) jointly lead the development of the cancer screening objectives and targets. The HP 2020 objectives for cancer, launched in December 2010 (3), were developed by federal experts with input from a diverse group of individuals and organizations, and were refined through a process of public comment and interagency review. Data from the 2008 National Health Interview Survey (NHIS) were used in setting targets for cancer screening, counseling about screening for cervical and breast cancer, and access to health care. Several targets were established to be a 10% improvement over the 2008 levels reported in the NHIS.

Reducing cancer illness and death is a key public health goal for the United States, and evaluating attainment of HP 2020 cancer-related objectives is an important gauge of our success in meeting this goal. We examine NHIS data from 2008 and 2010 to provide a benchmark for achieving HP 2020 objectives for 4 categories of cancer-related preventive health services: receipt of cancer screening tests, provider counseling about cancer screening, genetic counseling for women at high risk for breast or ovarian cancer, and access to health care. We include HP objectives and NHIS measures for health insurance status and having a source of ongoing medical care because receipt of cancer-related preventive services is strongly associated with these indicators of health care access (4–6). We also examine use of cancer-related preventive services by sociodemographic subgroups. Our overarching goal was to examine the most current NHIS data to derive insights into population-level challenges in meeting HP 2020 objectives for cancer-related preventive services.

## Methods

The NHIS is an in-person survey of a nationally representative sample of the US civilian noninstitutionalized population aged 18 years or older (7). Conducted annually by CDC’s National Center for Health Statistics (NCHS), the NHIS is one of the principal sources of information on the health status of Americans and their use of health services. To monitor national progress toward HP cancer goals, the Division of Cancer Control and Population Sciences, NCI, and the Division of Cancer Prevention and Control, CDC, have sponsored a Cancer Control Supplement to the NHIS, fielded every 5 years since 2000. Shorter supplements were fielded in 2003 and 2008. Table 1 provides the 2008 and 2010 NHIS sample sizes and corresponding US population denominators for each HP cancer control objective as well as the NHIS measure used to assess the objective. For the genetic counseling items, we used 2005 NHIS data instead of 2008 because questions about genetic counseling were not included in the 2008 NHIS. Complete NHIS survey instruments are available from NCHS (8). Response rates for the 2008 and 2010 NHIS were 63% and 61%, respectively, for the final samples, taking into account household nonresponse (9,10).

**Table 1:** Healthy People 2020 Objectives, Target Populations, Measures, and Estimates for Attaining Targets for Cancer Screening, Genetic Counseling, and Health Care Access, National Health Interview Survey (NHIS), 2008 and 2010

Healthy People Objective	Target Population	NHIS Measure	Target %	Baseline (2008 NHIS)		Interim (2010 NHIS)	
				% (95% CI)	Sample n (Population)	% (95% CI)	Sample n (Population)
**Cervical cancer screening**							
**C-15** Increase the proportion of women who receive a cervical cancer screening based on the most recent guidelines	Women aged 21–65 who have not had hysterectomy	Women aged 21–65 who have not had hysterectomy and had Pap testing in the past 3 years	93.0	84.4 (83.3–85.4)	7,560 (74,080,539)	82.9 (81.9–83.9)	9,073 (72,522,806)
**C-18.2** Increase the proportion of women who were counseled by their providers about Pap tests	Women aged 21–65 who have not had hysterectomy	Women aged 21–65 who have not had hysterectomy and were counseled by their providers about Pap testing	66.2	60.2 (58.7–61.7)	7,560 (74,080,539)	53.9 (52.6–55.3)	9,073 (72,522,806)
**Breast cancer screening**							
**C-17** Increase the proportion of women who receive a breast cancer screening based on the most recent guidelines	Women aged 50–74	Women aged 50–74 who had a mammogram in the past 2 years	81.1	73.7 (72.0–75.3)	4,237 (38,963,716)	72.4 (70.7–74.0)	5,336 (41,263,848)
**C-18.1** Increase the proportion of women who were counseled by their providers about mammograms	Women aged 50–74	Women aged 50–74 who were counseled by their providers about mammograms	76.8	69.8 (67.9–71.6)	4,237 (38,963,716)	59.5 (57.7–61.3)	5,336 (41,263,848)
**Colorectal cancer screening**							
**C-16** Increase the proportion of adults who receive colorectal cancer screening based on the most recent guidelines	Men and women aged 50–75	Adults aged 50–75 who had a blood stool test in the past 5 years, sigmoidoscopy in the past 5 years and blood stool test in the past 3 years, or colonoscopy in the past 10 years	70.5	52.1 (50.7–53.5)	7,776 (76,769,989)	59.1 (57.8–60.4)	9,782 (80,699,526)
**Prostate cancer screening**							
**C-19** (Developmental) Increase the proportion of men who have discussed with their health care provider whether to have prostate-specific antigen (PSA) testing	Men aged 50–74	Developmental	NA	NA	3,328 (35,971,417)	39.7 (37.7–41.7)	4,217 (37,771,177)
**Genetic counseling**							
**G-1** Increase the proportion of women with a family history of breast and/or ovarian cancer who receive genetic counseling	Women aged 18 or older who met USPSTF criteria, based on first-degree relatives, for BRCA1/2 genetic counseling referral and who do not have a personal history of breast or ovarian cancer	Women aged 18 or older who met the USPSTF criteria and who discussed having a genetic test for cancer risk with a health care provider	38.1	34.6 (18.2–51.1)^a^	143 (873,220)^a^	59.9 (34.0–71.9)	108 (849,185)
**Access to health services**							
**AHS-1.1** Increase the proportion of persons with health insurance coverage	People aged 0–64	People aged <65 who have any type of public or private health insurance coverage	100.0	83.2 (82.6–83.7)	65,758 (261,960,688)	81.8 (81.2–82.4)	79,536 (265,448,191)
**AHS-5.3** Increase the proportion of persons who have a specific source of ongoing care, adults aged 18–64 years	Men and women aged 18–64	Adults aged 18–64 who have a usual source of health care (other than an emergency department)	89.4	81.3 (80.4–82.2)	17,337 (187,950,006)	79.6 (78.8–80.4)	21,707 (190,813,389)
**AHS-5.4** Increase the proportion of persons who have a specific source of ongoing care, adults aged 65 years or older	Men and women aged 65 or older	Adults aged 65 or older who have a usual source of health care (other than an emergency department)	100.0	96.3 (95.6–96.8)	4,444 (37,277,310)	96.5 (95.9–97.1)	5,450 (38,691,705)

Abbreviations: CI, confidence interval; Pap, Papanicolaou; NA, not available; USPSTF, US Preventive Services Task Force.

a Baseline for genetic counseling is from the 2005 NHIS.

### Cancer control topics in the NHIS

**Cancer screening.** For cancer screening tests of interest, respondents were asked whether they ever had any of the tests and when they had their most recent one. Brief descriptions of each test were provided. We defined “receipt of breast, cervical, and colorectal cancer screening” as tests received consistent with age and screening interval recommendations (Table 1).

**Counseling for cancer screening.** We included counseling for several types of cancer screening. For men aged 40 years or older, the NHIS asks whether the respondent “discussed with a health care provider whether or not to have a PSA [prostate-specific antigen] test.” For other cancer screening tests, the survey asks whether a “doctor or health professional recommended” the test.

**Genetic counseling.** Adults who answered that they had heard of genetic testing were asked whether they “ever discussed the possibility of getting a genetic test for cancer risk with a health professional.” Responses to this question were analyzed for high-risk women, defined as those meeting the US Preventive Services Task Force criteria for receipt of genetic counseling for breast or ovarian cancer. Because the NHIS does not include information on Ashkenazi Jewish heritage or second-degree relatives, the criteria for classifying a woman as high risk were based only on information about cancer occurrence in first-degree relatives (11,12). The NHIS sample of these women was small: 139 in 2005 and 108 in 2010.

**Health insurance.** Respondents were categorized as having private or public health insurance or being uninsured. Individuals reporting no private insurance and Medicare, Medicaid, State Children’s Health Insurance Program (SCHIP), other public, other government, or military coverage were categorized as having public insurance.

**Has usual source of care.** Respondents who indicated that they had a place where they usually went when they were sick or needed advice about their health were defined as having a usual source of care. Those who reported they did not have a place or that the place they went to most often was an emergency department were defined as having no usual source of care.

**Race/ethnicity.** Respondents were characterized as non-Hispanic white, non-Hispanic black, Hispanic, non-Hispanic Asian, and other race/ethnicity (American Indian/Alaskan Natives and Native Hawaiian and other Pacific Islanders) (13).

**Income.** Annual income was recorded for each family and reported as a percentage of the federal poverty level (FPL). Families with income below 100% of the FPL are considered impoverished. Respondents were categorized according to their family income’s percentage of the FPL for the interview year: ≥400% FPL, 200%–399% FPL, 100%–199% FPL, and <100% FPL.

**Analysis.** Percentages and 95% confidence intervals were calculated using SUDAAN 10.0.1 (Research Triangle Institute, Research Triangle Park, North Carolina) to account for the NHIS’ complex sample design. We used *t* tests to determine whether differences from 2008 to 2010 estimates were significant. Multiple imputation was used to assign missing values for family income to appropriate categories, although results were similar if missing values were ignored in the analysis.

## Results

### Population receiving guideline levels of cancer screening tests

**Figure 1:**
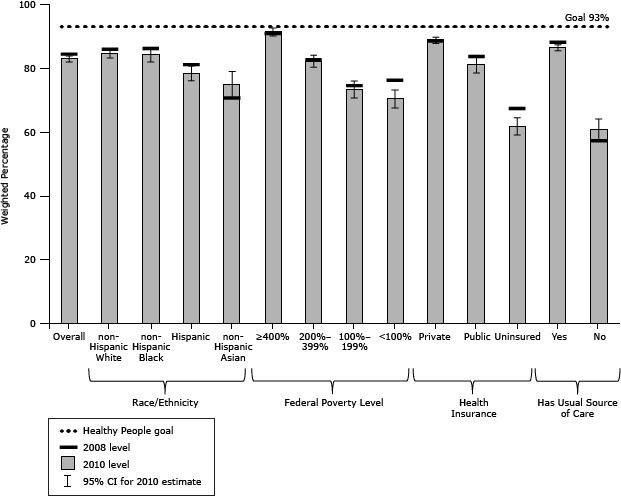
Women aged 21 to 65 years who had a Papanicolaou test for cervical cancer in last 3 years, National Health Interview Survey, 2008 and 2010. The Healthy People 2020 goal is 93%. Abbreviation: CI, confidence interval. **Table:** 

From 2008 to 2010, cervical cancer screening overall declined significantly by 1.5 percentage points (*P* = .0479) (Table 1) and small nonsignificant decreases were seen for most subgroups (Figure 1). Small increases occurred for 2 groups that experienced some of the lowest levels of screening in 2008: those without a usual source of care and non-Hispanic Asians. In 2010, the overall population, non-Hispanic whites, non-Hispanic blacks, those with private health insurance, and those with a usual source of care were within 10 percentage points of the HP 2020 target of 93.0%. Women in the highest income group, with income at or above 400% FPL, were 91.4%, within 2 percentage points of the target. Groups that were 20 percentage points or more below the target were the uninsured and those without a usual source of care.

Demographic Variables	2008	2010
	Weighted %	Weighted % (95% CI)
Overall	84.4	82.9 (81.9–83.9)
Race/ethnicity		
Non-Hispanic white	85.9	84.5 (83.2–85.7)
Non-Hispanic black	86.2	84.2 (81.9–86.2)
Hispanic	81.1	78.5 (76.1–80.7)
Non-Hispanic Asian	70.7	75.0 (70.6–79.0)
Ratio of family income to federal poverty level		
≥400% of federal poverty level	90.9	91.4 (90.1–92.6)
200%–399% of federal poverty level	82.6	82.3 (80.3–84.1)
100%–199% of federal poverty level	74.5	73.5 (70.7–76.0)
<100% of federal poverty level	76.2	70.5 (67.6–73.2)
Health insurance type		
Private	88.5	88.8 (87.7–89.7)
Public	83.6	81.2 (78.5–83.7)
Uninsured	67.4	61.8 (59.1–64.5)
Has usual source of care		
Yes	88.1	86.5 (85.5–87.4)
No	57.3	60.9 (57.6–64.1)

**Figure 2:**
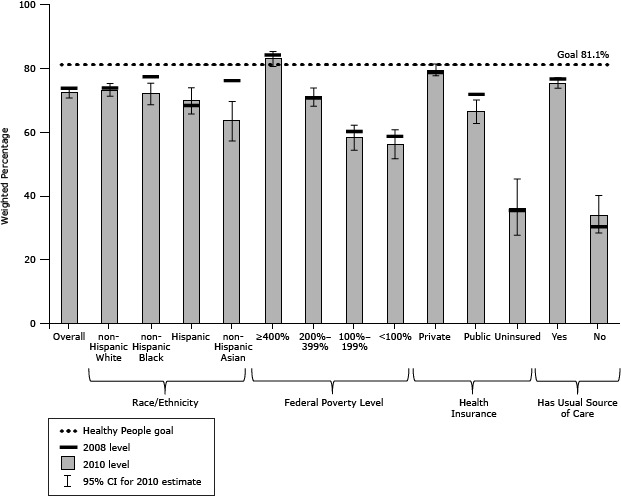
Women aged 50 to 74 years who had a mammogram in the past 2 years, National Health Interview Survey, 2008 and 2010. The Healthy People 2020 goal is 81.1%. Abbreviation: CI, confidence interval. **Table:** 

Breast cancer screening results were similar. Overall, the proportion of women reporting receipt of a mammogram from 2008 to 2010 declined nonsignificantly (Table 1). Non-Hispanic blacks, non-Hispanic Asians, and those with public health insurance all showed decreases, but these changes were not significant (Figure 2). In 2010, women in the highest income group exceeded the target of 81.1%. The overall population and 7 subgroups were within 10 percentage points of the target. Those who were uninsured, without a usual source of care, or whose incomes were less than 200% FPL were 20 percentage points or more below the target.

Demographic Variables	2008	2010
	Weighted %	Weighted % (95% CI)
Overall	73.7	72.4 (70.7–74.0)
Race/ethnicity		
Non-Hispanic white	73.8	73.2 (71.2–75.2)
Non-Hispanic black	77.3	72.1 (68.6–75.3)
Hispanic	68.3	69.9 (65.7–73.9)
Non-Hispanic Asian	76.1	63.6 (57.2–69.6)
Insurance type		
Private	78.6	79.5 (77.6–81.3)
Public	71.8	66.5 (62.7–70.1)
Uninsured	35.4	36.0 (27.7–45.3)
Ratio of family income to poverty level (imputed)		
≥400% of federal poverty level	84.1	83.0 (80.6–85.2)
200%–399% of federal poverty level	70.6	71.1 (68.1–73.8)
100%–199% of federal poverty level	60.2	58.3 (54.3–62.1)
<100% of federal poverty level	58.6	56.2 (51.6–60.7)
Have a usual source of care		
Yes	76.6	75.4 (73.7–77.0)
No	30.3	34.0 (28.4–40.1)

**Figure 3:**
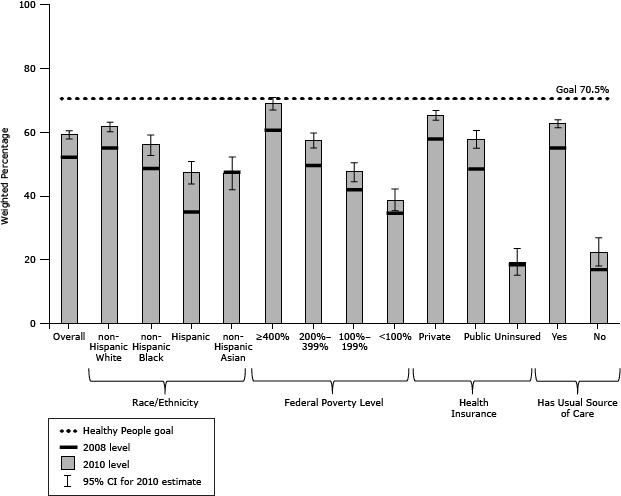
Adults aged 50 to 75 years who were screened for colorectal cancer, National Health Interview Survey, 2008 and 2010. Screening was defined as having had a home blood stool test in past year, sigmoidoscopy in the past 5 years and home blood stool test in the past 3 years, or a colonoscopy in the past 10 years. The Healthy People 2020 goal is 70.5%. Abbreviation: CI, confidence interval. **Table:** 

For colorectal cancer screening, a significant increase of 7 percentage points occurred for the overall population from 2008 to 2010 (Table 1). Moreover, screening increased significantly for all subgroups with the exception of non-Hispanic Asians and the uninsured (Figure 3). No group in 2010 met the target of 70.5%, but 4 subgroups (non-Hispanic whites, and those with incomes at or above 400% FPL, private health insurance, or a usual source of care) were within 10 percentage points of it. In contrast, Hispanics and non-Hispanic Asians and those without insurance, without a usual source of care, or with incomes less than 200% FPL were 20 percentage points or more below the target.

Demographic Variables	2008	2010
	Weighted %	Weighted % (95% CI)	
Overall	52.1	59.1 (57.7–60.4)	
Race/ethnicity			
Non-Hispanic white	55.0	61.6 (60.1–63.1)	
Non-Hispanic black	48.6	55.9 (52.7–59.1)	
Hispanic	34.9	47.2 (43.7–50.8)	
Non-Hispanic Asian	47.4	47.0 (41.9–52.2)	
Insurance type			
Private	57.8	65.3 (63.7–66.8)	
Public	48.4	57.7 (54.9–60.5)	
Uninsured	18.4	19.0 (15.2–23.5)	
Ratio of family income to federal poverty limit			
≥400% of federal poverty level	60.6	68.9 (66.9–70.8)	
200%–399% of federal poverty level	49.5	57.4 (55.0–59.7)	
100%–199% of federal poverty level	41.9	47.4 (44.5–50.4)	
<100% of federal poverty level	34.5	38.7 (35.3–42.2)	
Have a usual source of care			
Yes	55.0	62.6 (61.3–63.9)	
No	16.9	22.1 (18.0–26.9)

### Population receiving counseling about cancer screening and genetic testing

The 2008 NHIS asked women whether they had received a recommendation from a doctor for Papanicolaou (Pap) testing for cervical cancer and screening mammography and whether or not they were recently screened. In the 2010 NHIS, some respondents were skipped from this question if they gave certain answers to a question about why the test was not done. The question about why the test was not done was not included in the 2008 NHIS. By these measures, the proportion of women receiving a doctor’s recommendation for screening with the Pap test and mammography decreased measurably from 2008 to 2010 (Table 1), but the possibility that the decreases are an artifact of the change in the NHIS question cannot be ruled out. Therefore, we chose to focus on the 2010 estimates. 

Overall, 53.9% of women aged 21 to 65 years who reported receiving a Pap test in the past 3 years also reported receiving a doctor recommendation, compared with the target of 66.2% (Table 1). Subgroups within 10 percentage points of the target were non-Hispanic whites and those with health insurance, a usual source of care, and incomes at or above 400% FPL. Overall, 59.5% of women aged 50 to 74 years reported receiving a recommendation for a mammogram in the past 12 months, compared with the target of 76.8% (Table 1). Women with incomes at or above 400% FPL were the only subgroup within 10 percentage points of the target. Subgroups furthest from the targets for both Pap tests and mammograms were women with no insurance or no usual source of care.

Because counseling men about PSA testing was a developmental objective in HP 2020, no target was set. NHIS 2010 data indicate that 39.7% of men aged 50 to 74 years had received counseling about PSA testing (Table 1). Rates were slightly higher for those with private insurance or higher incomes. They were particularly low for those without health insurance or a usual source of care.

A total of 34.6% of women eligible for breast or ovarian cancer genetic counseling in 2005 and 59.9% in 2010 reported receiving provider counseling, compared with the target of 38.1% (Table 1). However, the 95% confidence intervals for the estimates are wide because of small sample sizes, so a trend toward increasing percentages from 2005 to 2010 cannot be confirmed.

### Population with access to health services

The HP 2020 objective for health insurance coverage is 100% for people under age 65. Overall, a nonsignificant decrease in health insurance coverage occurred from 2008 to 2010, from 83.2% to 81.8% (Table 1). Insurance coverage for most subgroups decreased slightly (Table 2). People with incomes at or above 400% FPL were the only subgroup within 10 percentage points of the target. Subgroups furthest from the target were Hispanics and those below 200% FPL.

**Table 2:** Proportion of People With Access to Health Care, by Population Subgroup, National Health Interview Survey (NHIS), 2008 and 2010

Demographic Characteristics	Adults Aged 18–64 y Who Have a Usual Source of Health Care (HP 2020 Target: 89.4%)		People Aged <65 y Who Have Health Insurance Coverage (HP 2020 Target: 100%)	
	2010, % (95% CI)	2008, % (95% CI)	2010, % (95% CI)	2008, % (95% CI)
**Race/ethnicity**				
Non-Hispanic white	83.3 (82.3–84.1)	84.9 (83.9–86.0)	86.3 (85.6–86.9)	87.5 (86.8–88.2)
Non-Hispanic black	77.0 (75.2–78.7)	78.4 (76.4–80.4)	79.7 (78.6–80.8)	82.1 (81.0–83.1)
Hispanic	66.1 (64.2–68.0)	67.3 (64.6–69.8)	68.0 (66.7–69.1)	66.7 (65.3–68.2)
Non-Hispanic Asian	79.4 (76.4–82.1)	81.9 (78.5–84.9)	83.6 (81.8–85.3)	86.8 (84.9–88.5)
**Ratio of family income to federal poverty level**				
≥400%	89.3 (88.3–90.2)	89.5 (88.4–90.5)	94.4 (93.9–94.8)	93.8 (93.3–94.3)
200%–399%	79.9 (78.5–81.1)	81.7 (80.2–83.1)	82.6 (81.7–83.5)	83.4 (82.5–84.4)
100%–199%	69.5 (67.6–71.3)	69.7 (67.4–72.0)	67.6 (66.3–68.9)	69.4 (68.0–70.7)
<100%	65.7 (63.5–67.8)	68.0 (65.2–70.6)	69.7 (68.3–71.2)	71.0 (69.3–72.8)
**Health insurance status**				
Private	89.2 (88.5–89.9)	89.7 (88.9–90.5)	NA	NA
Public	89.3 (88.0–90.4)	89.6 (87.8–91.1)	NA	NA
Uninsured	44.7 (42.8–46.5)	47.9 (45.8–50.0)	NA	NA

Abbreviations: HP, Healthy People; CI, confidence interval; NA, not applicable.

The target for having a usual source of care is 89.4% for people aged 18 to 64 years (Table 1). Overall, the proportion of the population reporting a usual source of care declined approximately 2% from 2008 to 2010, from 81.3% to 79.6%. Population subgroups meeting the target were those at or above 400% FPL and those with health insurance (Table 2). The overall population and several subgroups were within 10 percentage points of the target. Furthest from the target in both years were the uninsured.

## Discussion

We observed marked disparities in cancer screening and provider counseling rates for certain population subgroups, especially the uninsured and those with low income or no usual source of health care. Population subgroups whose access to care was the most compromised as measured by not having health insurance and not having a usual source of care included Hispanics and those below 200% FPL.

Given the well-established link between economic recession and decreased health insurance coverage (14), and the strong relationship between health insurance and cancer screening use, the gaps we observed for some HP measures may reflect the adverse state of the US economy during 2008 through 2010. However, neither Pap test nor mammography use increased during the past decade (15), suggesting that factors influencing cancer screening trends predate the most recent economic downturn and that meeting HP 2020 targets for cervical and breast cancer screening may be challenging. Further, our results show consistent and persistent disparities in receipt of cancer screening and provider counseling and in health care access for certain subgroups, suggesting that attaining HP cancer-related targets by 2020 may be challenging in the absence of new approaches to expand health insurance coverage, improve access to cancer screening and treatment services, better integrate clinical and community preventive services, and improve health literacy. 

One landmark development is the 2010 Patient Protection and Affordable Care Act; components of this national legislation are intended to reduce considerably the proportion of people who lack health insurance coverage or access to primary care providers or both. The importance of preventive health services for cancer control is recognized in the legislation, which makes certain services — including cervical, breast, and colorectal cancer screening — available with no cost-sharing in Medicare and in all new health insurance plans effective September 23, 2010 (16). Although newly eligible Medicaid beneficiaries also should experience improved access to these and other preventive services under health reform, some states may not provide equivalent coverage for existing Medicaid beneficiaries (17). It will be especially important to monitor attainment of HP objectives among health reform’s expanded Medicaid population. The legislation also calls for development of the National Prevention Strategy, which recommends improving integration of evidence-based clinical and community preventive services that may lead to an expanded role for public health in cancer screening (18–20). Finally, meeting HP objectives for counseling about cancer screening and genetic testing may require greater attention to provider-focused initiatives and interventions (21).

As the designated data source for evaluating many HP objectives, the NHIS has several strengths, including its large, nationally representative sample and high response rates. Limitations include the self-reported nature of the data; respondents may overestimate or underestimate cancer screening prevalence (22,23). The NHIS, though, is periodically revised to improve the description of screening tests and frame questions in a way intended to increase the accuracy of respondent reports. The NHIS also is limited in its ability to assess HP objectives for provider counseling about genetic testing and cancer screening. As previously mentioned, NHIS sample sizes of women at high risk for breast and ovarian cancer were too small for stable estimates of receipt of provider counseling for genetic testing. Regarding HP 2020 objectives for increasing the proportion of adults “counseled about cancer screening consistent with current guidelines,” the NHIS question about discussing with a health care provider whether to have PSA testing is descriptive of “counseling,” but the corresponding questions for cervical, breast, and colorectal cancer screening may be less so because they ask merely about doctor or health care provider “recommendation.” If broader issues of provider counseling for cancer screening tests, such as starting and stopping ages, differing screening intervals, or choice of screening tests, are incorporated into HP, the NHIS would need to be augmented. National surveys of health care providers — which NCI and CDC have supported for more than a decade, with several focusing on cancer screening (24–29) — might be an important, complementary data source to the NHIS.

Because cancer is a leading cause of premature death and a leading source of health care expenditures in the United States, HP goals for reducing cancer incidence and mortality are important for the public’s health and economic well-being. The NHIS is a key data resource for setting and evaluating HP objectives. Our assessment of the most currently available NHIS data suggests that meeting some cancer-related HP 2020 objectives may be feasible, but others — particularly those involving cancer screening and health care access — may depend on successful implementation of health reform provisions, better integration of clinical and community-based efforts to provide high-quality screening and treatment services, and elimination of health disparities in the United States.
